# Nutritional Genomics and the Mediterranean Diet’s Effects on Human Cardiovascular Health

**DOI:** 10.3390/nu8040218

**Published:** 2016-04-13

**Authors:** Montserrat Fitó, Valentini Konstantinidou

**Affiliations:** 1 Cardiovascular Risk and Nutrition Research Group (CARIN), CIBER de Fisiopatología de la Obesidad y Nutrición (CIBEROBN), Institut Hospital del Mar d’Investigació Mèdica (IMIM), Dr. Aiguader, 88, Barcelona 08003, Spain; 2 MEDOLIALI S.L. (DNANUTRICOACH^®^), Calle Diputación, 279, 1, 7, Barcelona 08007, Spain

**Keywords:** nutrigenetics, dietary pattern, prevention

## Abstract

The synergies and cumulative effects among different foods and nutrients are what produce the benefits of a healthy dietary pattern. Diets and dietary patterns are a major environmental factor that we are exposed to several times a day. People can learn how to control this behavior in order to promote healthy living and aging, and to prevent diet-related diseases. To date, the traditional Mediterranean diet has been the only well-studied pattern. Stroke incidence, a number of classical risk factors including lipid profile and glycaemia, emergent risk factors such as the length of telomeres, and emotional eating behavior can be affected by genetic predisposition. Adherence to the Mediterranean diet could exert beneficial effects on these risk factors. Our individual genetic make-up should be taken into account to better prevent these traits and their subsequent consequences in cardiovascular disease development. In the present work, we review the results of nutritional genomics explaining the role of the Mediterranean diet in human cardiovascular disease. A multidisciplinary approach is necessary to extract knowledge from large-scale data.

## 1. Introduction

### 1.1. Dietary Patterns

Food patterns refer to the consumption of differing amounts, proportions, and combinations of diverse foods and beverages, and the variations in the frequency of their intake. The relevance of overall high-quality food patterns should be taken into consideration as the synergies and cumulative effects among different foods and nutrients are behind their health benefits [1]. A single-variable approach has been traditionally followed in nutritional studies. One consequence of this has been to promote debate among proponents of single-nutrient causes and solutions to diet-related health problems [2]. Diets are more than the sum of their components, and this has been a fundamental concern with the one-variable-at-a-time (OVAT) approach due to the fact that the multidimensional essence of nutrition is not captured. Moreover, diets form only part of a healthy lifestyle recommended for the treatment of numerous complex and multifactorial diseases such as cardiovascular ones. Dietary patterns are a major environmental factor that people are exposed to numerous times a day during their lives. Diet is also the environmental factor that people can learn to control from early on to prevent diet-related diseases and promote healthy living and aging.

A healthy dietary pattern, such as the Mediterranean diet, can be a useful and complementary tool to better control classic cardiovascular risk factors within the frame of lifestyle recommendations. In this regard, the first step in hypertension and other cardiovascular risk factors management is to follow a healthy diet, such as the traditional Mediterranean diet [3] or the DASH (Dietary-Approaches-to-Stop-Hypertension) diets [4]. Both diets are characterised by foods rich in phytochemicals, such as fruit and vegetables, which have been inversely associated with high blood pressure and hypercholesterolemia, among other cardiovascular risk factors.

However, the specific characterization of a dietary pattern remains a challenge because it is hampered by the complexity of interpreting multidimensional dietary data. Interactions between diet and the human genome have led to intense research and debate regarding the effectiveness of personalized nutrition as a more adequate tool to prevent chronic diseases than the traditional one-size-fits all recommendations [5]. The nutritional genomics field, although still in its early stages, offers encouraging results for its widespread incorporation into clinical practice.

### 1.2. The Mediterranean Dietary Pattern

The traditional Mediterranean diet refers to dietary patterns found in olive-growing areas of the Mediterranean region since the 1960s [6]. It is considered a single entity consisting of diet-variants from each region in the Mediterranean basin. It is based on an abundant and daily consumption of olive oil, which is the main source of fat, and is characterized by: (a) high intake of vegetables, fruit, legumes, whole grains, nuts, and seeds; (b) frequent (and moderate) intake of red wine with meals; (c) moderate consumption of seafood, fermented dairy products (cheese and yogurt), poultry, and eggs; and (d) low consumption of red meat, meat products, and sweets. Moreover, the Mediterranean dietary pattern also encompasses daily physical activity, proper hydration (approximately 2 L of water a day) and social eating habits [7]. Olive oil is considered a hallmark of this dietary pattern, resulting in high intakes of monounsaturated fatty acids (MUFA) and lower intakes of saturated fatty acids (SFA).

The Mediterranean diet may not be markedly different from other recommended diets worldwide but its basic element, olive oil, makes it unique and contributes an additional value to its health benefits [8]. To the best of our knowledge, the Mediterranean diet is the only dietary pattern that, to date, has been investigated in depth and that is why the present review is based on this dietary pattern. Current knowledge from observational studies supports its instrumental role in the context of cardiovascular disease (CVD) prevention. As yet, few randomized, dietary intervention trials have been performed assessing long-term effects of a diet intervention, with a solid design, in primary and secondary prevention. The Lyon Diet Heart Study reported the benefits of a Mediterranean-type dietary pattern on the secondary prevention of CVD with 605 volunteers who had suffered a first myocardial infraction [9]. The protective effect of the diet was maintained up to 4 years after the first infarction. The most relevant impact of the traditional Mediterranean diet (TMD) as a primary protection against cardiovascular endpoints has only recently been described in the PREDIMED (PREvencion con DIeta MEDiterranea) study [10,11]. The PREDIMED study was a randomized, controlled and large-scale (*n* = 7447) intervention trial which tested the long-term effects (5-year follow up) of the TMD on incident cardiovascular diseases. The PREDIMED study reported, for the first time, high-level evidence of the primary prevention for cardiovascular events such as a hard composite endpoint (myocardial infarction, stroke, and cardiovascular mortality) [10,12], stroke [10], atrial fibrillation [13], type-2 diabetes [14], and peripheral vascular disease [15]. In addition, an improvement of the classic cardiovascular risk factors, at the medium and long term, in high-risk individuals was described after adherence to the traditional Mediterranean diet.

Traditionally the healthy benefits of the Mediterranean diet have been attributed to its richness in antioxidants. Antioxidant compounds can exert their beneficial effects through chemical reactions, once incorporated into the organism, but also during the digestion of the diet components. The anti-inflammatory effects in addition to the antioxidant ones, the formation of nitrolipid compounds, the viability of the cell-membranes and monolayers, and the modification of the microbiota can be other remarkable mechanisms. Furthermore, the mechanism of action of antioxidants, and other nutrients, is very much related to their capacity to modulate gene and protein expression.

### 1.3. Methods

Our aim is to present evidence concerning the well-researched Mediterranean dietary pattern included in nutritional genomics studies. A literature review was performed in MEDLINE up to July 2015. The search aimed to identify current knowledge on nutritional genomics mechanisms that could explain the beneficial effects of the Mediterranean diet on preventing cardiovascular diseases. The following Medical Subject Heading Terms (MESH) were used: Mediterranean diet, humans, nutritional genomics, nutritigenomics, nutrigenetics, dietary pattern, epigenomics, interventions, studies. In the present review, we revised more than 70 English articles. Those describing nutritional genomics mechanisms, after adherence to the Mediterranean dietary pattern, in human intervention studies were finally included. Nutritional genomics mechanisms and studies on issues other than the Mediterranean dietary pattern were not the objective of the present review.

## 2. Nutritional Genomics Mechanisms

**Figure 1:**
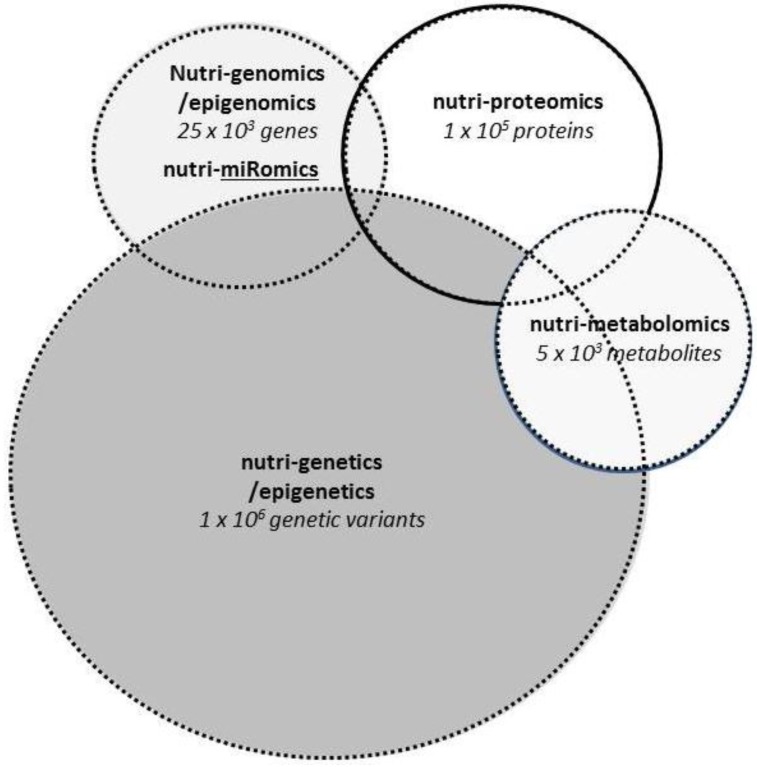
Proposed nutritional genomics mechanisms. The “Nutri-” prefix in the name of each of the 4 categories defines the influence of nutrition on genomics/epigenomics, proteomics, metabolomics and genetics/epigenomics.

Our genetic predisposition is responsible for a percentage of CVD risk that varies among people. Genetic predisposition could explain a great part of the differential responses observed in individuals after the same dietary treatments, and could help health professionals personalize even more their recommendations. Nutritional genomics has emerged as a relatively new field of research assessing the mechanisms by which nutrients and dietary patterns interact with our genome at different stages. It embraces a systems biology approach to evaluate individual risk factors in the light of genetic diversity at the transcriptome, genetic, metabolome, and epigenome levels (Figure 1) [16].

The integration of data from all these areas of complexity needs advanced computational methodology to establish networks of interacting biological components and determine linked activities. The goal of nutritional genomics is to take into consideration the complexity of dietary patterns, culture, and metabolic processes that lead to health or disease, and to provide personalized and precise recommendations to prevent common, multifactorial diseases decades before their clinical manifestation.

To the best of our knowledge, conclusive evidence for the effects of interactions between genes and dietary patterns on cardiovascular health is lacking. Genome-wide interaction studies (GWIS) are a real possibility in order to identify specific gene-diet interactions even though providing the necessary sample size presents some difficulty [17]. We review here the current results of nutrigenetics, nutrigenomics, nutri-metabolomics, nutri-epigenomics, nutri-epigenetics, and nutri-miRomics related to Mediterranean diet interventions in humans. Table 1 summarizes the relevant studies. The majority of these studies include mainly Spanish populations and have been performed in the framework of PREDIMED study. We strongly encourage the design and performance of similar studies, in terms of design and follow-up duration, in different populations, to further confirm current results.

### 2.1. Nutrigenetics

Nutrigenetics aims to gain greater understanding of the mechanisms associated with inter-variability among individuals and to develop more personalized dietary recommendations. Genetic predisposition in humans is arising as a main factor to identify sub-groups that would benefit from dietary interventions. There is increasing evidence that individuals with different genotypes respond differently to diet. Genotyping methods detect the most common form of genetic variants, called single nucleotide polymorphisms (SNPs), in the human DNA sequence. The SNPs constitute the genetic fingerprint of each person, and genome-wide association studies (GWAS) assess the relationships between SNPs and concrete observable traits (phenotypes) [18].

The PREDIMED results support the notion that individual genetic predisposition toward CVD risk could be influenced by dietary components, mainly by a stricter adherence to the Mediterranean diet pattern. Corella *et al.* studied the genetic predisposition to present increased fasting glucose, total cholesterol, LDL cholesterol, triglyceride (TG) concentrations, and stroke incidence in homozygote individuals for the T allele of the rs7903146 polymorphism in the TCF7L2 (transcription factor 7-like 2) gene. They found that this predisposition could be attenuated by adherence to the Mediterranean diet [19].

Other genetic variants were also found to be protective. This is the case of the rs3812316 functional single nucleotide polymorphism (SNP) at the MLXIPL (Max-like protein X interacting protein-like) gene [20]. This SNP has been associated with lower systemic TG concentrations. Using data from the PREDIMED study, Ortega-Azorin replicated previous associations with TG concentrations and showed that they were modified by adherence to a Mediterranean diet. Thus, the potential CVD protection was enhanced in individuals who had higher adherence to the Mediterranean diet. Most importantly, the reduction in CVD risk that was associated with the Mediterranean diet, as shown in the whole PREDIMED study [10], was significantly enhanced in carriers of the minor, G allele, at this MLXIPL locus.

Recently, leukocyte telomere length, considered as a potential biomarker of biological age, has also been described as being involved in the gene-diet interaction in the PREDIMED study [21]. In this work, DNA from 521 participants of the PREDIMED Study was genotyped for the presence of the Ala allele in rs1801282 SNP of the PPARγ2 (peroxisome proliferator-activated receptor gamma) gene. It was observed that the presence of the Ala allele prevented telomere attrition associated with aging. Higher adherence to the Mediterranean dietary pattern showed benefits after a 5-year follow-up in subjects with the Ala allele because they had longer telomeres. The authors concluded that this gene-diet interaction (rs1801282-Mediterranean diet) could have an added value in the improvement of personalized dietary recommendations based on genetic predisposition to achieve healthy aging and lower CVD risk.

Garcia-Rios *et al.* have proposed that dietary recommendations in individuals with metabolic syndrome may also require a more personalized approach [22]. They have studied genetic variation at the CLOCK (circadian locomotor output cycles kaput) gene and their data support the hypothesis that a habitual consumption of a healthy diet, such as the Mediterranean one, could contribute to triggering glucose metabolism through interactions with the rs1801260 SNP in the CLOCK gene. Lopez-Guimera *et al.* have analyzed the role of emotional eating behavior and the interaction of the same CLOCK 3111 T/C polymorphism on the effectiveness of a weight-loss program. In a 30-week follow-up with a Mediterranean population, they reported that the CLOCK 3111 T/C SNP interacted with emotional eating behavior to modulate total weight loss [23].

Glycaemia, lipid profile, stroke incidence, telomeres length, and emotional eating behavior are some of the characteristics that have been seen to be affected by genetic predisposition and adherence to the Mediterranean diet. Thus, taking into account individual genetic make-up can be useful in the management and control of these traits and their subsequent consequences in CVD development.

### 2.2. Nutrigenomics

Nutrition researchers have acknowledged that gene–diet interactions could play a major role in the development of and protection against chronic degenerative diseases [24]. Although the mechanisms behind statistically significant associations and interactions remain unclear, given the continuous novel knowledge being obtained about the genome [36], some of the ambiguities are being resolved. Nutrients may influence gene expression directly as ligands for nuclear receptors or indirectly by inducing epigenetic modifications without changing the underlying DNA sequence. Gene expression changes are measured in the level of messenger RNA (mRNA) by transcriptomic techniques [37] such as, but not limited to, microarrays and real-time reverse transcription PCR [38].

The high antioxidant content of the Mediterranean diet could be one of its protective mechanisms of action. Antioxidants have the capacity to modulate gene, protein expression [25] and subsequently, metabolite production [29]. Oxidation and inflammation are intertwined processes, and when sustained for a long period may be involved in the physiopathology of many diseases [39]. Indeed, chronic inflammation is another key patho-physiological factor in the development of obesity, type-2 diabetes, and CVD. Previous nutrigenomic intervention studies have shown that the Mediterranean diet pattern has a protective effect on the expression of pro-atherosclerotic genes involved in vascular inflammation, foam cell formation, and thrombosis [24,25,26]. In addition, adherence to a Mediterranean dietary pattern may protect against artery wall production of inflammatory mediators [26,27]. Moreover, recent nutrition research has indicated that our diet is able to influence, directly or indirectly, the immune system and well-being, and thus affects inflammatory disease development [40,41,42].

In an overall transcriptomic study within the PREDIMED study, the principal pathways in the physiopathology of cardiovascular events, such as atherosclerosis, renin-angiotensin, nitric oxide and angiopoietin signaling, were modulated by TMD + EVOO, whereas hypoxia and eNOS signaling pathways were modified by TMD enriched with EVOO or nuts [28]. After simultaneous testing adjustment, 9 pathways were modulated by TMD + EVOO and 4 by TMD+Nuts while none of the pathways changed in the control group. Thus, a higher overall modulation of gene expression was observed after adherence to a Mediterranean diet. In this regard, it can also be considered as a better adaptive response to detrimental factors, such as a mechanism for hormesis [43].

Gene expression changes have been reported in humans, both after the Mediterranean diet and olive oil consumption at postprandial or sustained time [24]. Changes in the regulation of gene expression are reflected in fingerprinting patterns that could prove very useful for the future development of biomarkers. However, caution should be exercised due to heterogeneity of the studies assessing gene expression changes in human tissues and body fluids. Further research on larger population sizes, with robust designs and standardized methodologies, is required before definite nutrigenomic-based recommendations can be formed.

### 2.3. Nutri-Metabolomics

Metabolomics aims to study the entire small molecule (metabolite) complement of a system. Metabolites are defined as possessing an atomic mass inferior to 1.5 kDa and can be exogenous, endogenous, or derived from microbiome metabolism. A huge variety of metabolites exists, including peptides, lipids, nucleotides, carbohydrates, amino acids, and carbohydrates [44,45].

The effects of an intervention based on the Mediterranean dietary pattern were assessed by Bondia-Pons *et al.* [29]. They used liquid chromatography coupled to the quadrupole-time of a flight-MS (LQ-QTOF/MS) metabolic profiling technique to screen plasma from individuals with MetS features who participated in the Metabolic Syndrome Reduction Study in Navarra (RESMENA), a randomized, controlled trial. It was reported that a two-month pattern based on the Mediterranean diet produced significant changes in plasma metabolic profile. However, and in spite of the fact there were associations between metabolic and clinical variables, these changes disappeared after six months, suggesting that compliance had declined during the self-control period.

Nutri-metabolomic effects were also assessed within the frame of the PREDIMED study. Metabolomics results enabled the classification of individuals according to their specific food consumption or dietary patterns. Vazquez-Fresno *et al.* studied the metabolomics pattern of non-diabetic participants in the PREDIMED study after one and three years of follow-up [30]. The Proton nuclear magnetic resonance (^1^H NMR) urinary metabolome profile was analyzed in the participants who were classified as consuming either a low fat diet or a Mediterranean one. Results showed that the most prominent hallmarks in the groups that followed the Mediterranean diet were related to the metabolism of carbohydrates (3-hydroxybutyrate, citrate, and cis-aconitate), creatine, creatinine, amino acids (proline, *N*-acetylglutamine, glycine, branched-chain amino acids, and derived metabolites), lipids (oleic and suberic acids), and microbial co-metabolites (phenylacetylglutamine and p-cresol). In addition, the dietary walnut fingerprinting by an HPLC-q-ToF-MS untargeted metabolomics approach has been assessed [31]. Walnut consumption was characterized by 18 metabolites, such as markers of fatty acid metabolism, ellagitannin-derived microbial compounds, and intermediate metabolites of the tryptophan/serotonin pathway. Likewise, the urinary metabolome signature of cocoa intake in PREDIMED participants has been reported [32].

Metabolomic profile has been also characterized in the elderly. Gonzalez-Guardia *et al.* investigated the effect of the Mediterranean diet when supplemented with coenzyme Q10 in elderly men and women after 4 weeks of intervention [33]. Greater hippurate urine levels were described after the Mediterranean diet + CoQ and higher phenylacetylglycine levels after saturated fat diet consumption in women. These results suggest that the long-term consumption of a Mediterranean diet supplemented with Q10 could exert benefits on healthy aging and on the prevention of processes related to chronic oxidative stress.

Gut microbiota play a key role in CVD; having being identified as a possible novel CVD risk factor, they represent a realistic therapeutic target [46]. The microbiome-gut-brain axis links human physiology (and pathology) to millions of other microorganisms that share the same host. The microbiome is affected by dietary intake [47] and specific dietary factors (carbohydrate, protein, and Mediterranean foods) seem to regulate CVD risk factors through the modulation of microbial populations and activities [48].

Microbiota also appear to play a critical role in the clock–nutrition interplay. Our circadian clock is a highly specialized and stratified network that drives and orchestrates proper rhythms for organism homeostasis. It operates as a critical interface between nutrition and homeostasis. The circadian clock is a mechanism calling for more attention concerning the beneficial effects of dietary patterns on disease prevention and health maintenance; in addition, the disruption of the circadian rhythm may cause manifestations of metabolic syndrome [49].

Metabolomics results that could serve as future biomarkers of consumption and/or adherence are increasing. Moreover, nutri-metabolomics studies could offer another level of knowledge for nutritional personalization based on our gut microbiota although, once again, further studies are necessary before solid conclusions can be reached.

### 2.4. Nutri-Epigenomics and Nutri-Epigenetics

Epigenetics studies the heritable DNA modifications able to regulate chromosome architecture and modulate gene expression without changing the underlying sequence. Epigenetic phenomena are critical for the aging process that takes place from embryonic development to later adult life. Increasing evidence supports the complex interactions that exist among food components, dietary patterns, and epigenetic modifications such as histone modifications, DNA methylation, non-coding RNA expression, and chromatin remodeling factors. Such alterations are among the hallmarks of age-related chronic inflammatory diseases including metabolic syndrome and diabetes [50]. In analogy to GWAS studies, epigenome-wide association studies (EWAS) have arisen [51]. The first results demonstrate the central role of epigenomic information and of epigenetic changes in response to diet and environmental conditions to understand human disease. Nutrition in early life can induce long-term changes in DNA methylation thus affecting individual susceptibility to a range of diseases [52].

Nutritional epigenetics is a novel mechanism underlying gene-diet interactions. Epigenetic phenomena are critical for the aging process, from embryonic development to later adult life, and the complexity of integrating all these data is a huge multidisciplinary challenge.

### 2.5. Nutri-miRomics

Nutrimiromics has emerged as a subsidiary field of nutritional genomics assessing how nutrients affect microRNAs (miRs) and their function. MiRs are small non-coding RNA sequences of single sequences of 19-24 nucleotides located in intra- or inter-regions of protein coding genes [53]. MiRs have emerged as the major regulators of a great number of physiological processes including cell differentiation, apoptosis, and lipid metabolism, and they have been reported to regulate glucose homeostasis, β-cell function, and insulin signaling. The main function of miRs is silencing their target genes by acting as specific inhibitors of mRNA [54]. The complementarity between miRs and mRNA is not always perfect and, depending on the grade of complementarity, miRs could induce mRNA degradation, de-adenylation, and transductional repression [55].

Numerous SNPs in miRs-target sites have also been demonstrated to have allele-specific effects. For example, the minor allele of the SNP rs13702 in the 3′ untranslated region (3′ UTR) of the lipoprotein lipase (LPL) gene disrupts a miRs recognition element seed site for the human miRNA-410, resulting in a gain-of-function and lower plasma TG [34]. This work has revealed that the molecular basis for some of the observed cardiovascular effects could involve differential regulation by miRs [34]. Corella *et al.* reported a novel association between a microRNA target site variant and stroke incidence, modulated by a high-unsaturated-fat Mediterranean diet in terms of decreasing TG levels and possibly stroke risk in rs13702 C allele carriers, within the frame of the PREDIMED Study [35]. MicroRNAs have emerged as relevant epigenetic regulators in cardiovascular diseases. The study of miRNA target site polymorphisms as functional variants could contribute to a better understanding of the mechanisms of physio-pathology of CVD, among others.

## 3. Conclusions

The interactions among food and nutrients, calorie intake, meal frequency and timing, single-nutrient modifications, microbiota, and genetic predisposition in modulating the key mechanisms that maintain cellular tissue and organ function during aging are not yet well-established. However, individual genetic variation should definitely be taken into account before developing personalized recommendations. The Mediterranean dietary pattern, which is more than just a diet, is to date the most studied at the level of evidence-based medicine. Nutritional genomics embraces and researches different aspects of nutrition-related molecular mechanisms at all levels of systems biology approach such as transcriptomics, genomics, genetics, and metabolomics. Although the benefits of the traditional Mediterranean diet in the prevention of cardiovascular disease have been assessed, the nutritional genomics mechanisms are yet to be fully elucidated. Data reported in the present review has not yet been fully replicated in different ethnic populations, ages, genders, and physiopathological conditions. The nutritional genomics field is quickly advancing but a great deal of effort is yet required from the scientific community, in different disciplines, to establish the subjacent mechanisms linked to the nutritional genomics of the healthy benefits of Mediterranean diet on cardiovascular diseases. The integration of OMICS, both at a single-OMICS level or preferably at a multi-OMICS one, will be crucial to better understand the inter-individual mechanisms behind the conferred protection of the traditional Mediterranean diet as well as to gain greater knowledge with respect to personalized nutrition. An integrative approach is necessary to extract conclusions from large-scale data. Further nutritional genomics research, at all levels, will be necessary to evaluate optimal “doses” of this dietary pattern, from an early stage *in utero* gestation until later adult life, to control diet-related disease, and to promote healthy living and aging.

## Figures and Tables

**Table 1:** Nutrigenetics, nutrigenomics, nutri-metabolomics, and nutri-miRomics studies related to Mediterranean diet interventions in humans.

Nutrigenetics	SNPs Tested	Outcome	Reference
	rs7903146 (homozygotes for the T risk allele) at the TCF7L2 gene.	Increased fasting glucose, total cholesterol, LDL-C, TG, stroke incidence	[19]
	rs3812316 (carriers of the G protective allele) at the MLXIPL gene.	Lower TG, reduction in CVD risk	[20]
	rs1801282 (carriers of the Ala-G protective allele) at the PPARγ2 gene.	Higher adherence to the Mediterranean diet strengthens the prevention of telomere shortening	[21]
	rs1801260 in the CLOCK gene (homozygous for the major T allele).	Triggering glucose metabolism in patients with metabolic syndrome	[22]
	rs1801260 in the CLOCK gene (carriers of the minor C allele)	Less weight loss for the C carriers with high emotional score (emotional eaters).	[23]
**Nutri-Genomics**	**Gene Expression**	**Gene groups Affected**	
	Protective modulation (*i.e.*, ADRB2, IL7R, IFNγ, MCP1, TNFa *etc.*).	Pro-atherosclerotic in vascular inflammation, foam cell formation, thrombosis, oxidative stress	[24,25,26]
	Protective modulation (*i.e.*, IFNγ, ARHGAP15, IL7R, POLK, ADRB2 *etc.*).	Artery wall production of inflammatory mediators	[26,27]
	Canonical pathways modulation.	Atherosclerosis, hypertension, renin-angiotensin, nitric oxide, angiopoietin signaling, hypoxia, eNOS signaling pathways	[28]
**Nutri-Metabolomics**	**Sample Type/Population Characteristics**	**Results**	
	Plasma from individuals with MetS.	altered metabolic profile	[29]
	Urine from non-diabetic adults.	Classification of individuals by evaluating changes in the urinary metabolome at different time points	[30]
	Spot urine samples of free-living population.	Predictive model of dietary walnut exposure	[31]
	Urinary metabolome in free-living population.	Improved predictive model of dietary exposure to cocoa by combining different metabolites as biomarkers	[32]
	Urinary metabolome in elderly men and women.	Greater hippurate after Med+CoQ and higher phenylacetylglycine levels after SFA diet in women	[33]
**Nutri-miRomics**	**MicroRNA/Target SNP**	**Effect**	
	miRNA-410/rs13702 in the 3′untranslated region (3′UTR) of the lipoprotein lipase (LPL) gene.	Disruption of the recognition element seed site, gain-of-function, and lower TG.	[34]
	miRNA-410/rs13702 C allele carriers.	Stroke incidence modulated by diet by decreasing TG and stroke risk after a high-unsaturated fat Mediterranean diet	[35]

ADRB2: adrenoreceptor beta 2, surface; ARHGAP15: Rho GTPase activating protein 15; CLOCK: circadian locomotor output cycles kaput; CVD: cardiovascular disease; IFNγ: interferon gamma; IL7R: interleukin 7 receptor; LDL-C: low density lipoprotein cholesterol; MCP1: chemokine (C-C motif) ligand 2; MetS: Metabolic Syndrome; MLXIPL: Max-like protein X interacting protein-like; POLK: polymerase (DNA directed) kappa; PPARγ2: peroxisome proliferator-activated receptor gamma; TCF7L2: transcription factor 7-like 2; TG: triglycerides; TNFα: tumor necrosis factor α.
